# Hamstrung but Not Out: A Track Star’s Sprint Through a Rare Tendon Tear

**DOI:** 10.7759/cureus.89544

**Published:** 2025-08-07

**Authors:** Suleman Janjua, Andrew Lew, James Chin, Alex Pallas, Nicholas Moore

**Affiliations:** 1 Sports Medicine, Ascension Providence Hospital, Southfield, USA; 2 Family Medicine, University of Nevada, Reno, Reno, USA; 3 Sports Medicine, Zucker School of Medicine at Hofstra, New York City, USA; 4 Interventional Radiology, Enloe Medical Center, Chico, USA

**Keywords:** hamstring, musculoskeletal, pain, semtendinosus, tear, track

## Abstract

Hamstring injuries are common among athletes; however, complete tears of the semitendinosus are rare. Given the infrequency of these injuries, there is no consistent treatment algorithm. For athletes pursuing conservative, non-surgical management, physical therapy and rest may facilitate shorter recovery times. We present the case of a collegiate track athlete with no prior surgeries or hamstring injuries who successfully returned to sport following conservative treatment of a distal semitendinosus tear. To our knowledge, this represents the first reported case of successful return to play following nonoperative management of such an injury.

## Introduction

The hamstring muscle group consists of the biceps femoris, semimembranosus, and semitendinosus [1]. Hamstring injuries are typically classified based on their location, either proximal, mid-substance, or distal. Additionally, the extent of the tear, partial or complete, and the number of muscles or tendons involved further help to determine the severity of the injury.

Hamstring injuries account for approximately 30% of all sports-related muscle injuries [2]. Of these, 32-37% involve the semitendinosus, most commonly affecting the proximal tendon [3,4]. In contrast, distal semitendinosus injuries are rare, even among elite athletes, and complete distal avulsions are exceedingly uncommon [5,6]. These injuries are more frequently seen in sports that involve sudden acceleration, deceleration, or hyperextension of the knee, such as sprinting or soccer, and may be associated with nearby ligamentous injuries [5,7,8].

This report highlights a rare case of an isolated complete distal avulsion of the semitendinosus tendon.

## Case presentation

A 23-year-old male collegiate track athlete presented with posterior left knee pain and swelling, primarily localized to the medial hamstring insertion. The symptoms began after he felt a distinct "pop" while running. He described the pain as both dull and sharp, rated 5/10 at rest, and noted that it worsened with running and knee flexion. He denied numbness, tingling, skin discoloration, or temperature changes. He had no prior surgeries, was not on any medications, and had no history of injury to the left lower extremity or any relevant family medical history.

On examination, his back revealed no tenderness to palpation. The patient demonstrated a full range of motion and 5/5 strength in all lower extremity muscle groups. Ligamentous testing of the left knee showed no laxity with varus or valgus stress at both 0° and 30° and was negative for anterior drawer, posterior drawer, Lachman, pivot shift, both medial and lateral McMurray, and Thessaly tests. The examination of the right knee was unremarkable.

On the left, the pes anserine bursa, medial collateral ligament (MCL), lateral collateral ligament (LCL), medial and lateral joint lines, and Gerdy's tubercle were all non-tender. However, there was localized tenderness over the hamstring insertion and in the popliteal fossa. Neurologic examination was normal, including negative straight leg raise and slump tests. Deep tendon reflexes were 2+ at both the patellar and Achilles tendons.

**Figure 1:**
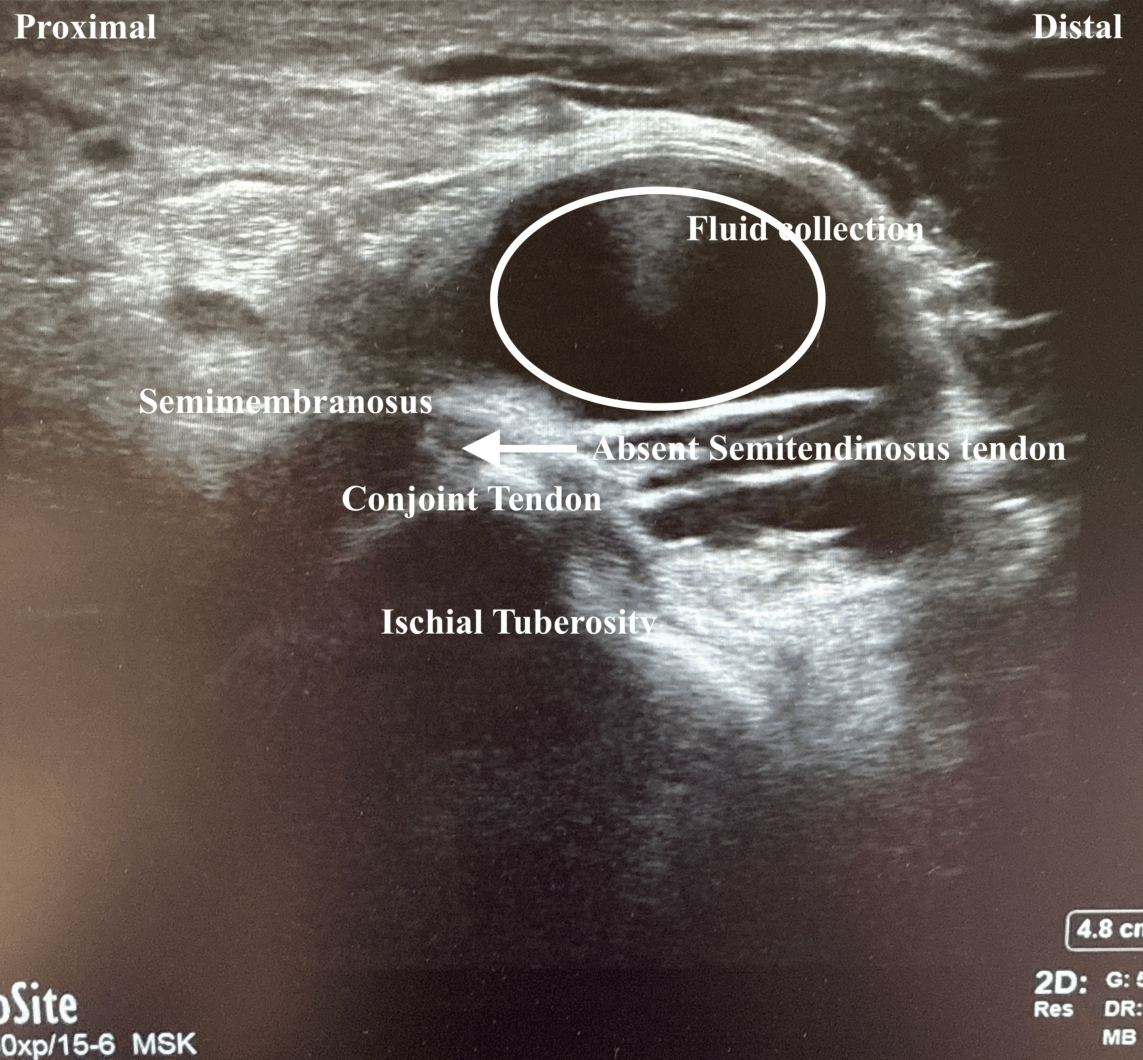
Labeled ultrasound long-axis view of the knee. Note the fluid collection (circle) and absent semitendinosus tendon (arrow).

**Figure 2:**
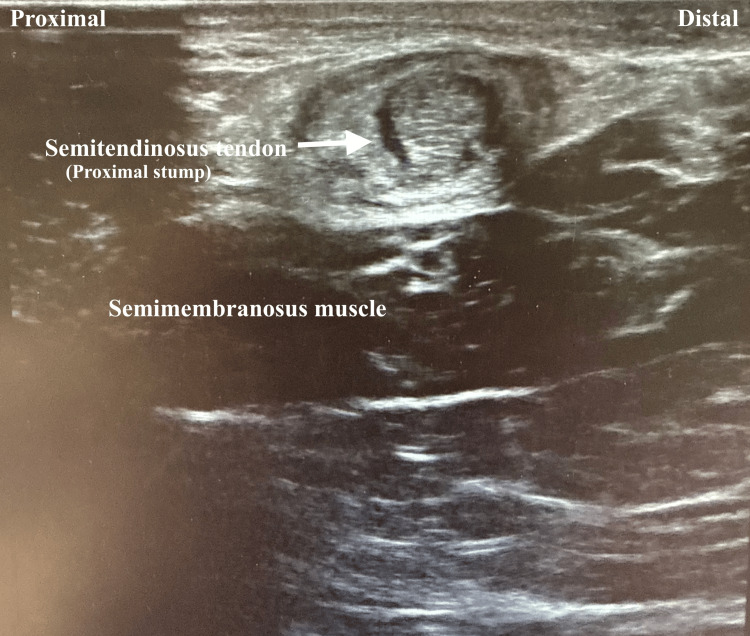
Short axis view of ultrasound of knee. The semitendinosus is showing a stump with surrounding fluid suggestive of tendon retraction (arrow).

**Figure 3:**
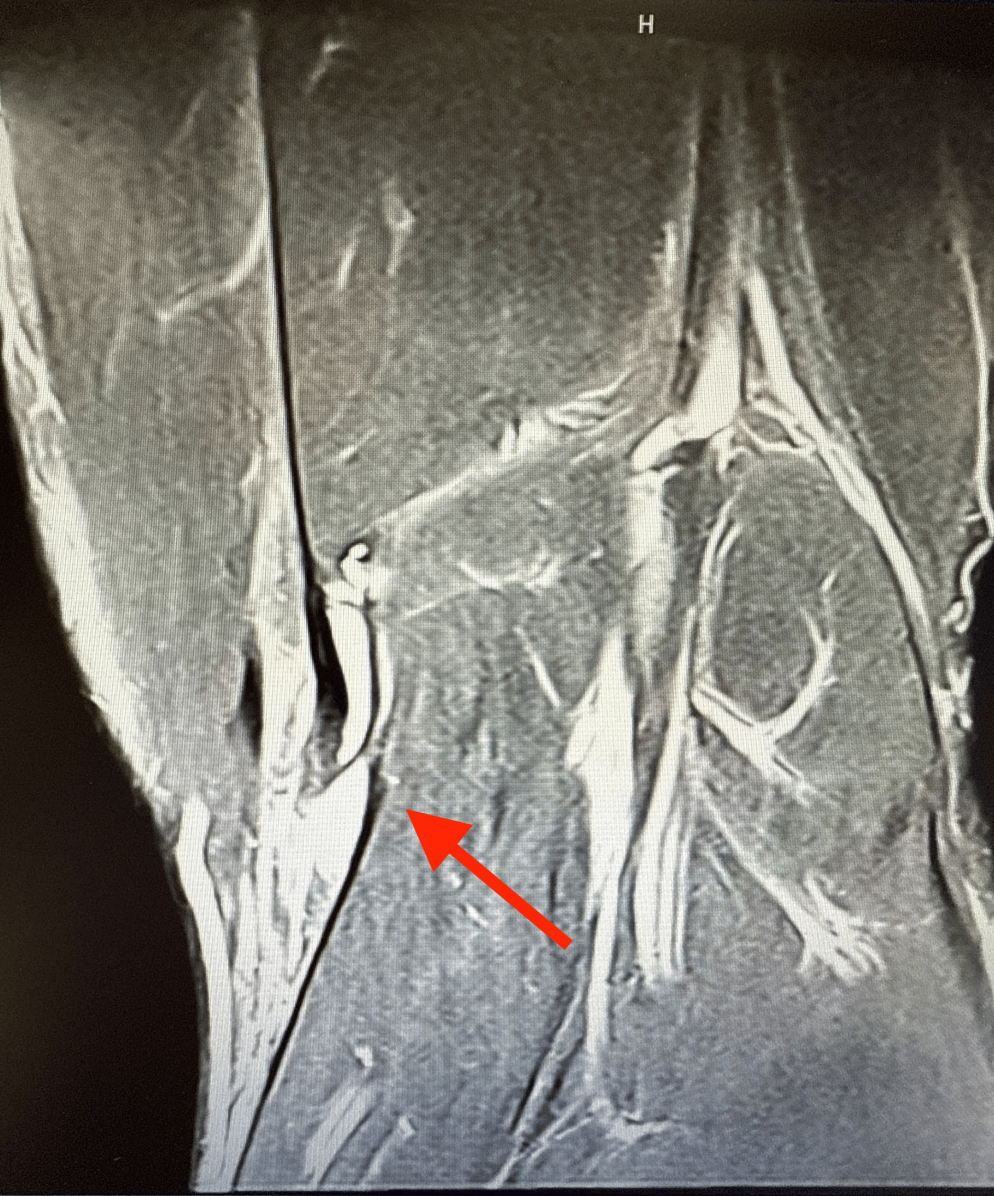
MRI of the knee showing discontinuity of the semitendinosus tendon with the surrounding fluid pocket (arrow).

**Figure 4:**
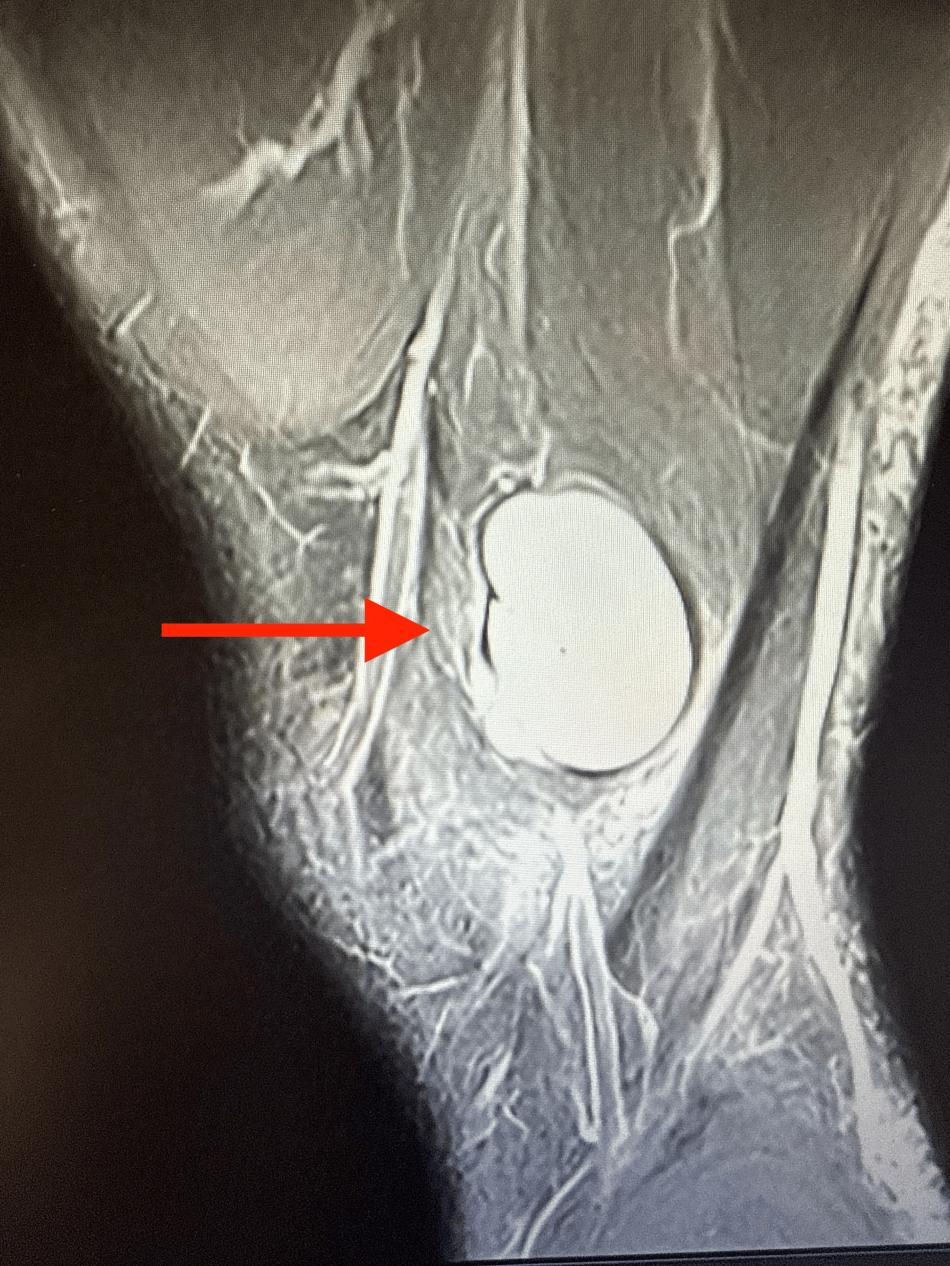
MRI of the knee showing hyperdensity with sharp demarcating edges suggesting fluid collection in the distal thigh (arrow).

**Figure 5:**
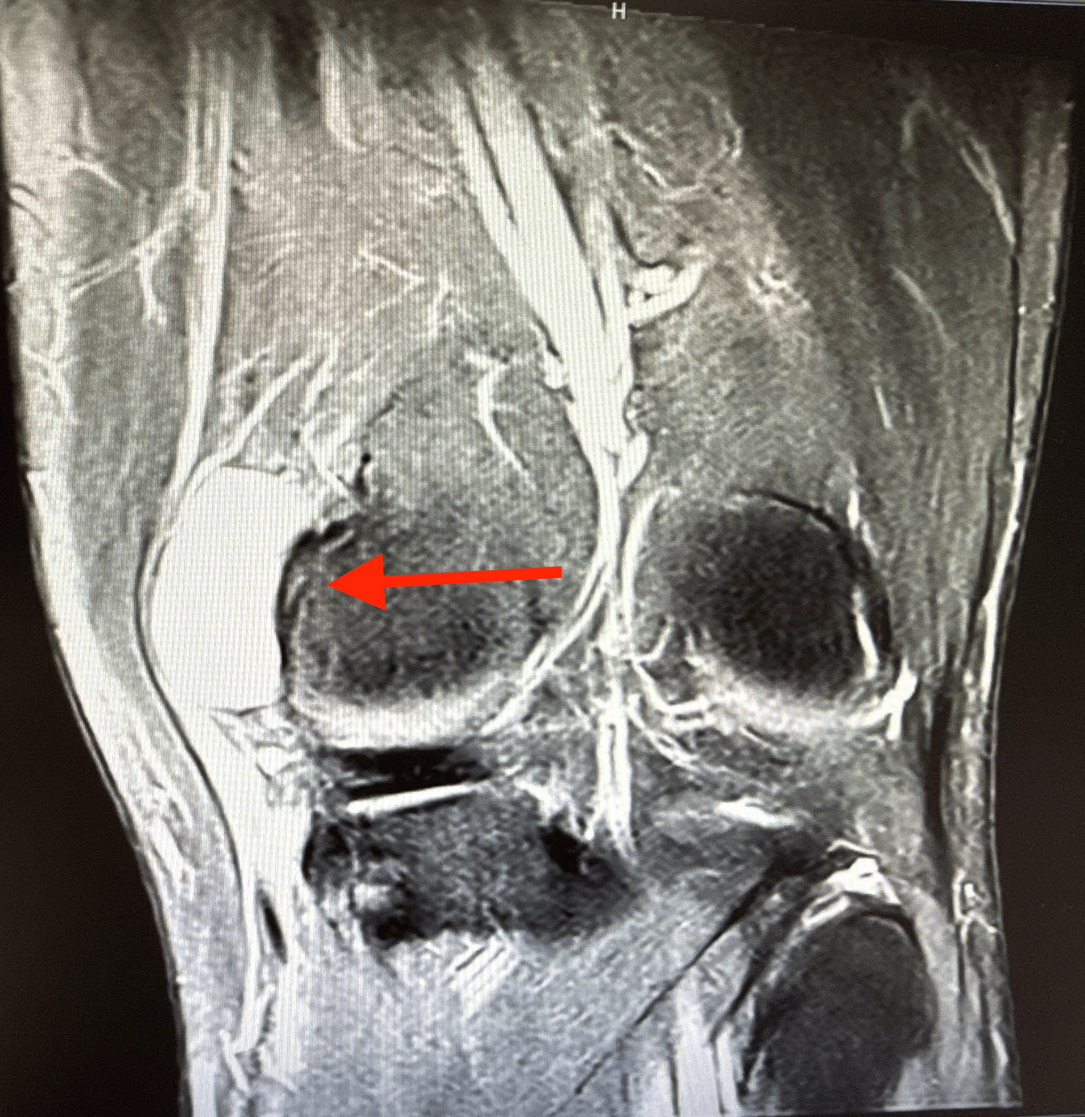
Coronal MRI of the knee with hyperdensity, which suggests fluid collection (arrow).

**Figure 6:**
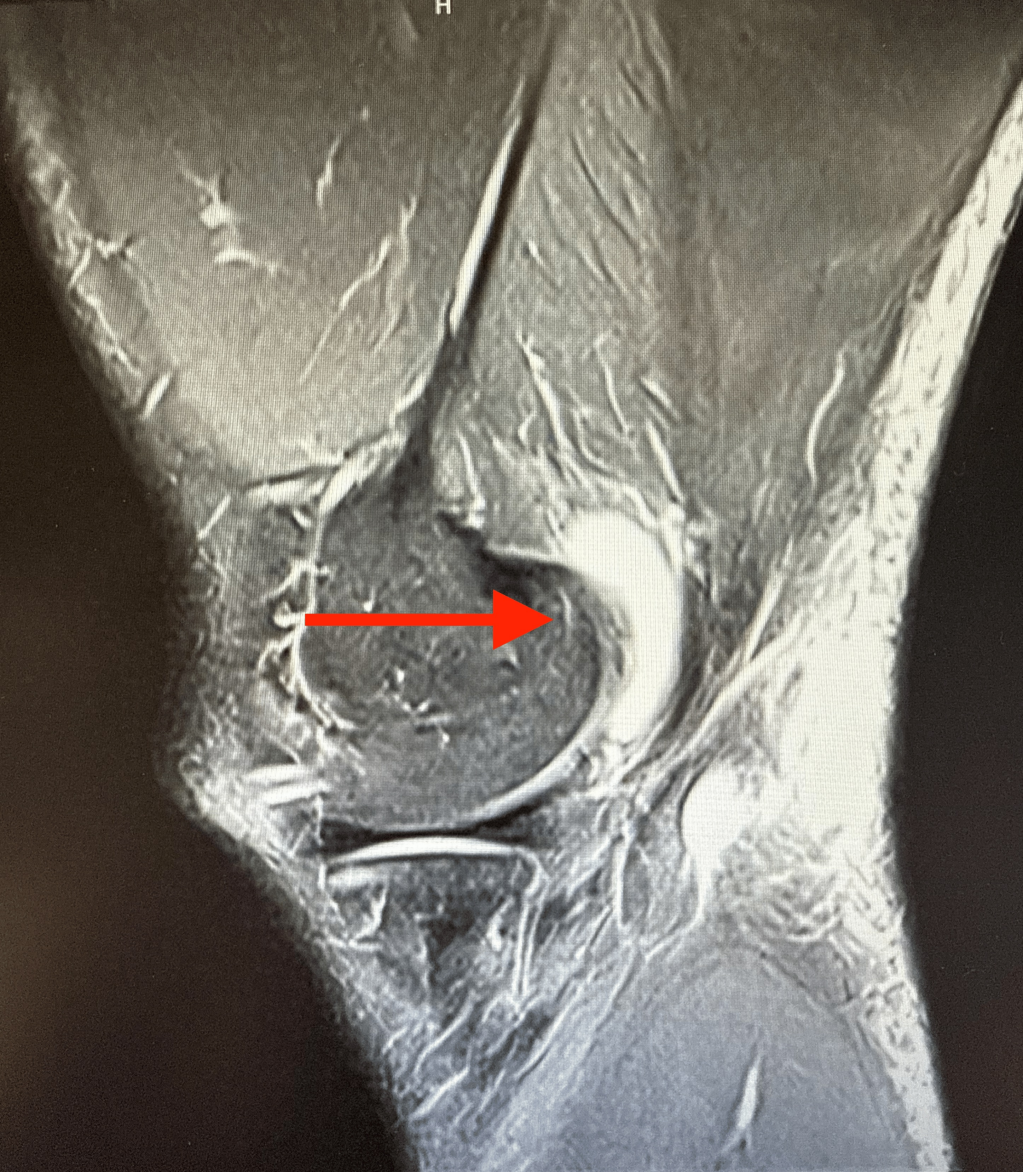
Sagittal MRI showing the position of the wound relative to the distal portion of the semitendinosus at the level of the knee. Collection of fluid and discontinuation of the tendon (arrow).

Four-view radiographs of the left knee revealed normal alignment, with no evidence of fracture, degenerative changes, or soft tissue abnormalities. Ultrasound of the left lower extremity demonstrated a full-thickness tear of the semitendinosus tendon, with a large hematoma in the hamstring musculature and a retracted tendon stump at the distal margin of the hematoma (Figures 1, 2). A minimal Doppler signal was present. These findings were visualized in both sagittal and transverse planes. A subsequent MRI of the left knee confirmed a full-thickness, full-width tear of the distal semitendinosus tendon at its attachment on the medial proximal tibia (Figures 3-6).

The patient was referred to physical therapy with a focus on hamstring rehabilitation and was advised to follow up after 4 to 6 weeks. He was counseled that if symptoms worsened or if follow-up imaging failed to show appropriate healing, orthopedic surgical consultation would be considered. At follow-up, his symptoms had resolved, and he was cleared to return to sport as tolerated.

## Discussion

Distal semitendinosus tendon tears typically result from high-stress activities such as sprinting, sudden directional changes with the foot planted, knee hyperextension, jumping, or direct trauma [5,6,9]. Distal injuries are generally larger than those affecting the proximal tendon or muscle belly. Patients often describe a sudden “pop” in the back of the knee, followed by swelling and delayed pain or tenderness in the lower posterior thigh.

The risk of a distal semitendinosus tear increases in individuals with a history of injury to the region, including prior tendon harvesting for anterior cruciate ligament (ACL) reconstruction [3]. Magnetic resonance imaging (MRI) is the diagnostic modality of choice, allowing for precise assessment of the tear’s location and severity, as well as differentiation between tendon and muscle belly injuries [2]. MRI also helps identify which specific hamstring tendon is involved.

Among 23 reported cases of isolated complete distal semitendinosus avulsions or tears, approximately 70% were treated surgically [8]. Patients undergoing conservative management typically recovered in about 1.5 months, whereas those who underwent surgical repair required an average of three months for recovery [8]. However, surgical intervention was often required later due to complications [4,8,9]. Due to the rarity of distal semitendinosus injuries, no standardized treatment protocol currently exists [7,9]. In this case, physical therapy was trialed. Additionally, although not utilized in this case, adjunctive therapies such as platelet-rich plasma (PRP) injections, shockwave therapy, red light therapy, and other alternative modalities may also confer therapeutic benefit. We report a patient motivated to trial nonoperative treatment with successful outcomes and achieving fast recovery timelines to return to sport.

## Conclusions

Complete isolated tears of the distal semitendinosus are rarely reported in the literature, and no standardized treatment protocol currently exists. While prior cases have described initial conservative management, many were complicated by poor outcomes and ultimately required surgical revision. To the best of our knowledge, this case represents the first documented instance of successful conservative treatment for a complete distal semitendinosus tear.
